# Alkaline Phosphatase Electrochemical Micro-Sensor Based on 3D Graphene Networks for the Monitoring of Osteoblast Activity

**DOI:** 10.3390/bios12060406

**Published:** 2022-06-13

**Authors:** Ning Zhao, Jiaci Shi, Ming Li, Pengcheng Xu, Xuefeng Wang, Xinxin Li

**Affiliations:** 1 Department of Orthodontics, Shanghai Ninth People’s Hospital, Shanghai Jiao Tong University School of Medicine, College of Stomatology, Shanghai Jiao Tong University, National Center for Stomatology, National Clinical Research Center for Oral Diseases, Shanghai Key Laboratory of Stomatology, Shanghai 200011, China; zhaon1995@126.com; 2 College of Chemistry and Materials Science, Shanghai Normal University, Shanghai 200234, China; 1000495035@smail.shnu.edu.cn; 3 State Key Lab of Transducer Technology, Shanghai Institute of Microsystem and Information Technology, Chinese Academy of Sciences, Shanghai 200050, China; liming01@mail.sim.ac.cn (M.L.); xpc@mail.sim.ac.cn (P.X.); 4 University of Chinese Academy of Sciences, Beijing 100049, China

**Keywords:** alkaline phosphatase (ALP), electrochemical sensor, three-dimensional graphene networks, osteoblast cells, screen-printed electrode

## Abstract

Alkaline phosphatase (ALP) is a significant biomarker that indicates osteoblast activity and skeletal growth. Efficient ALP detection methods are essential in drug development and clinical diagnosis. In this work, we developed an in-situ synthesized three-dimensional graphene networks (3DGNs)-based electrochemical sensor to determine ALP activity. The sensor employs an ALP enzymatic conversion of non-electroactive substrate to electroactive product and presents the ALP activity as an electrochemical signal. With 3DGNs as the catalyst and signal amplifier, a sample consumption of 5 μL and an incubation time of 2 min are enough for the sensor to detect a wide ALP activity range from 10 to 10,000 U/L, with a limit of detection of 5.70 U/L. This facile fabricated sensor provides a quick response, cost-effective and non-destructive approach for monitoring living adherent osteoblast cell activity and holds promise for ALP quantification in other biological systems and clinical samples.

## 1. Introduction

Monitoring osteoblast activity is crucial for the diagnosis of bone diseases or evaluation of therapy efficacy. Bone alkaline phosphatase (ALP) is one of the basic enzymes in osteoblasts and plays a critical role in bone mineralization [1,2]. Bone ALP is highly expressed in the cells of mineralized tissue and can be released into the circulation; therefore, the level of bone ALP increases when osteoporosis or some other metabolic bone diseases occur [3,4]. The total serum bone ALP has been considered a bone biomarker in clinics, but the detection of bone ALP is challenging because it presents at a very low level and exists in a complex biological environment [5,6]. Therefore, sensitive, rapid, and on-site monitoring methods for bone ALP are highly demanded in biomedical research and clinical diagnosis.

To date, various detection strategies have been developed for measuring ALP activity, such as colorimetry [7,8], surface-enhanced resonance Raman scattering [9,10], and fluorescence assay [11,12,13]. Despite their reliability and sensitivity, many of the testing methods are time-consuming, requiring professional operators and sophisticated instruments. In this context, electrochemical sensors offer an alternative due to their high sensitivity, fast response, easy to use, portability, and low cost. Direct electrochemical tests of ALP activity involve the enzymatic hydrolysis of the phosphoric acid esters substrate to electroactive phenolate products, which can be subsequently electrochemically oxidized to quinone compounds. The electrochemical signal generated from phenolate product oxidation is proportional to the ALP activity [14,15,16], so the performance of the sensor is closely associated with its ability to measure the ALP enzymatic products. 

Many efforts have been made to achieve high sensitivity; for example, some nanomaterials such as nanoceria particles [17] and copper sulfide nanoparticles [18] were used as catalysts and signal amplifiers for the substrate-based electrochemical ALP sensors. Among various nanomaterials applied to electrochemical sensors, graphene performs a specific activity for the oxidation of ALP enzymatic phenol products due to its high electrical conductivity and π-electron system [19,20]. However, the strong van der Waals forces and inter-sheet junction contact resistance between graphene sheets make them aggregate or restack easily, resulting in a diminished surface area and electron diffusion rate [21,22], which deteriorate the sensor performance. Three-dimensional graphene networks (3DGNs) in various porous forms are emerging members of the graphitic family. Combining 3D porous structures and excellent intrinsic properties of graphene, 3DGNs exhibit features such as large surface areas, high pore volumes, good mechanical strengths, and quick mass and electron transport rates, which can accelerate electrolyte movement and analyte access to the sensing interface [23,24,25,26]. Compared with their counterparts, such as graphene quantum dots and 2D graphene materials [27,28], these advantages make 3DGNs as promising carbon nanomaterials in electrochemical sensors.

**Figure 1:**
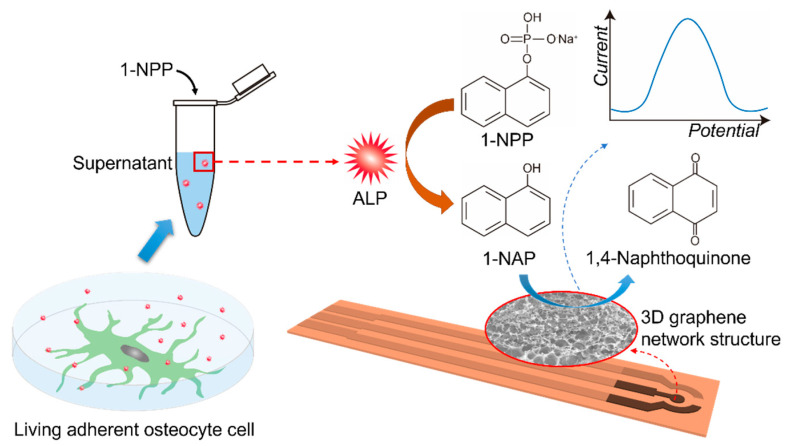
Detecting ALP secretion activity of living adherent osteocyte cells using supernatant by a 3DGNs modified SPE-based sensor.

Herein, an ALP activity electrochemical sensor is fabricated with a 3DGNs modified SPE electrode. The 3DGNs were prepared by a one-step in-situ method and used as a catalytic amplifier for electrochemical detection of ALP activity. The resulting 3DGNs have a large surface area, high conductivity, and good electrocatalytic activity. As shown in Figure 1, ALP hydrolyzes the non-electroactive substrate 1-naphthyl phosphate (1-NPP) to enzymatic product 1-naphthol (1-NAP), which can be further oxidized to 1,4-naphthoquinone and generate an electrochemical signal. The synergetic effects from π–π conjugate and electrochemical accumulation of 3DGNs can facilitate the 1-NAP oxidation process. After optimizing the pH value and incubation time, the sensors were examined by testing ALP activity in osteoblastic cells supernatant. Unlike some previously reported destructive methods which measured ALP from the cell lysates [29,30,31], our non-destructive approach can monitor ALP activity by obtaining culture supernatants of living adherent cells. This type of sensor provides a solution for enhancing the detection sensitivity of ALP activity by using a facile and cost-effective procedure based on the 3DGNs nanomaterials. 

## 2. Materials and Methods

### 2.1. Materials

Alkaline phosphatase (activity 10 units/mg) was purchased from Sangon Biotech (Shanghai). Diethanolamine (DEA), anhydrous magnesium chloride, penicillin-streptomycin, β-glycerophosphate disodium salt hydrate, and ascorbic acid were provided by Sigma-Aldrich. 1-Naphthol and 1-naphthyl phosphate were purchased from Shanghai Macklin Biochemical. α-MEM was from Hyclone, Cytiva. Interferon-gamma recombinant mouse protein (IFN-γ) was provided by Gibco. Fetal bovine serum (FBS) was purchased from Atlanta Biologicals. Phosphoric acid and potassium chloride was purchased from Shanghai Chemical Reagent Corp. All chemicals were of analytical reagent grade and used without further purification. Deionized water was used for all solution preparation. 

### 2.2. In-Situ Growth of 3DGNs on Screen-Printed Electrodes

Screen-printed electrodes (SPE) were batched-fabricated on the polyimide (PI, 175 μm thickness) substrate consisting of a silver-silver chloride (Ag-AgCl) reference electrode, a carbon counter electrode, and a 3DGNs-functionalized working electrode. The 3DGNs (~1.0 mm diameter) were in-situ synthesized on PI substrate by a laser direct-writing technology (Speedy 100R, Trotec, Bunkyō, Tokyo). The power and scanning speed of the CO_2_ laser (10.6 μm) were set as 7.5 W and 20 cm/s, respectively.

### 2.3. 3DGNs Nanostructure and Electrochemistry Characterization 

The morphologies of 3DGNs were characterized by scanning electron microscopy (SEM, S4800, Hitachi, Chiyoda City, Tokyo). The microstructures were analyzed by transmission electron microscopy (TEM, Tecnai G20, FEI) operating at 200 kV. Raman spectrum was obtained by an inVia Reflex confocal Raman microscope (RENISHAW) with a 514-nm laser. Cyclic voltammetry (CV) and differential pulse voltammetry (DPV) detection were carried out by a CHI 660E electrochemical workstation. The DPV curves were obtained with the following parameters: amplitude of 0.05 V, pause width of 0.06 s, sampling width of 0.02 s, and pulse period of 0.5 s. Baseline correction for DPV was done by measuring a control sample.

### 2.4. ALP Enzymatic Product Detection

For the electrochemical measurements of ALP-catalyzed products (1-NAP), a 1-NAP standard stock solution of 10 μM was prepared by dissolving the 1-NAP powder in DEA buffer (10 mM DEA, 0.5 mM MgCl_2_, 0.1 M KCl). A series of 1-NAP concentrations of 10 nM, 100 nM, 1 μM, 3 μM, 5 μM, and 7 μM were diluted from the stock solution with DEA buffer to obtain the calibration curve. The DEA buffer was taken as a control sample. Solutions with each concentration were measured three times. The electrochemical measurements were carried out by dropping 5 μL of 1-NAP solution within the working area of the electrode, then DPV tests read the oxidation current. Each 3DGNs/SPE was used only once to prevent fouling after the oxidation of 1-NAP.

### 2.5. ALP Activity Detection

ALP powder was dissolved and diluted with DEA buffer. The ALP activity test was performed by adding 5 μL ALP solution containing ALP with a particular activity to 45 μL of 500 μM 1-NPP in DEA buffer. The solution was then incubated at 37 °C in a water bath for a certain reaction period. An aliquot of 5 μL was sampled onto the 3DGNs/SPE. 

For sensor calibration, a 10,000 U/L stock solution was prepared and diluted with DEA to different ALP activities of 10, 100, 600, 1000, 4000, and 6000 U/L. DEA buffer was taken as a control sample. Each ALP sample (5 μL) was mixed with 45 μL of 500 μM 1-NPP at 37 °C for 2 min, followed by 10 μL of 2 M phosphoric acid. Finally, 5 μL of the mixed solution was taken for the DPV test on 3DGNs/SPE. The solution with each ALP activity was measured three times. 

### 2.6. Cell Culture and ALP Activity Measurement in Cell Supernatant

An immortalized cell line IDG-SW3, which could mimic Osteoblast-to-Late-Osteocyte differentiation, was used in this study. α-MEM supplemented with heat-inactivated 10% FBS and 100 U/mL (100 ug/mL) penicillin-streptomycin were utilized as the general medium. A culture medium of 2500 U of IFN-γ to 100 mL was used as the cell proliferation medium.

Cells were plated in the proliferation medium at 33 °C at an initial density of 4 × 10^4^ cells/cm^2^. After reaching 100% confluence in 2–3 days, cells were changed to a 37 °C incubator and the general medium containing 4 mM β-glycerophosphate and 50 mg/mL ascorbic acid, without IFN-γ, was changed for differentiation. 

For supernatant analysis, IDG-SW3 cells in the proliferation stage were seeded with densities of 0.45 × 10^5^, 0.90 × 10^5^, and 1.80 × 10^5^ per well for differentiation, respectively. The ALP activities in supernatants were detected after 24 h of incubation directly from the differentiation medium.

For the electrochemical tests, 5 μL of the cell culture supernatant was incubated for 2 min with 45 μL of 500 μM 1-NPP in DEA buffer at 37 °C, then 10 μL of 2 M phosphoric acid was added as the stop solution. A 5 μL aliquot of the mixed solution was taken for the DPV test on 3DGNs/SPE. The differentiation medium was used as a control sample. 

## 3. Results and Discussion

### 3.1. Characterization of the In-Situ Synthesized 3DGNs

**Figure 2:**
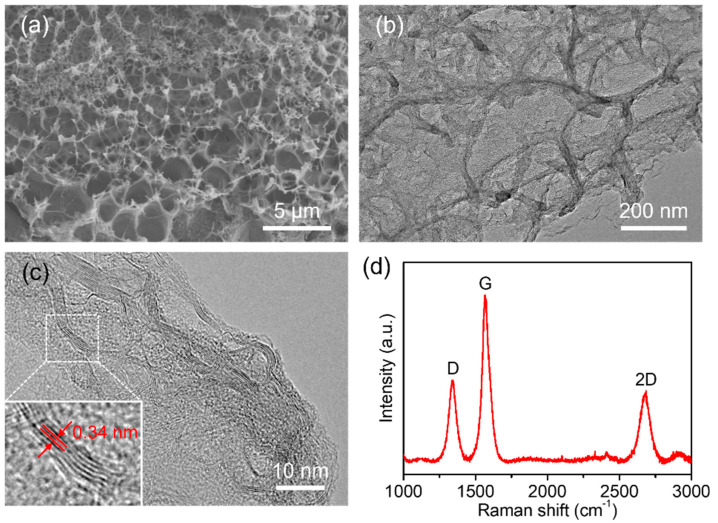
Characterization of 3D graphene networks in-situ grown on PI substrate. (**a**) SEM and (**b**) TEM images showing the 3D network structure. (**c**) HRTEM image indicating the few-layer structure and interplanar distance of graphene. (**d**) Raman spectrum confirming the successful growth of graphene.

3DGNs can be directly synthesized on the working electrode through a laser direct-writing method, as the PI substrate serves as a carbon source. SEM and TEM images in Figure 2a,b show the 3D porous network structure of the nanomaterial. The high-resolution TEM (HRTEM) image in Figure 2c also proves the successful growth of multilayered graphene, as the lattice fringe spacing of 0.34 nm is associated with the interplanar distance of the (002) crystal plane of graphite. Furthermore, the few-layer (3–10-layer) structures in Figure 2c indicates that the as-synthesized 3DGNs are graphene-based nanocomposite structures rather than graphite films [28]. The distinct 2D peak at ~2689 cm^−1^ in the Raman spectrum in Figure 2d further confirms the synthesized graphene. The clear D peak suggests the presence of large amounts of defects in the laser-induced 3D porous graphene networks. Using the methylene blue adsorption method as detailed in Supplementary Materials, the surface area of 3DGNs is measured to be 448.73 m^2^/g, which is in accordance with previous reports [28]. Owing to the porous network structure, the 3DGNs grown in-situ with a larger surface area can adsorb more analytes and provide more active sites for electrochemical catalysis. In addition, the 3D porous structure can effectively avoid graphene aggregation or restack.

### 3.2. Electrochemical Detection of ALP Enzymatic Product

**Figure 3:**
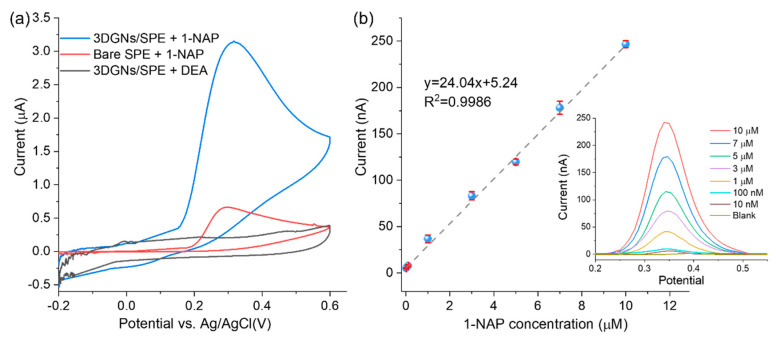
(**a**) CV characterization of the electrochemical oxidation of 1-NAP by 3DGNs/SPE compared with bare SPE; (**b**) calibration curve of the current peaks for 1-NAP oxidation at 0.35 V. Error bars represent the RSD of triple measurements. The inset shows DPV curves of the enzymatic product 1-NAP in DEA buffer with the concentration range from 0.01 to 10 μM obtained by 3DGNs/SPE.

As illustrated in Figure 1, 1-NAP is the ALP enzymatic product and can be oxidized to generate the electrochemical signal. Thus, the electrochemical sensing response to 1-NAP can be used to represent the ALP activity. Before ALP activity measurements, the sensing response to 1-NAP should be investigated. In order to demonstrate that the oxidation signal of 1-NAP can be amplified by 3DGNs material, CV tests were carried out and the results are shown in Figure 3a. When scanning from −0.2 V to 0.6 V, 1-NAP was oxidized to 1,4-naphthoquinone. As shown by the red curve in Figure 3a, an oxidation peak occurred at 0.35 V with a current of 0.66 μA on the bare SPE. In contrast, the oxidation peak current obtained from 3DGNs/SPE was 3.13 μA (blue curve), which was about five-fold greater than that of the bare SPE. The signal amplification may result from the large π conjugated structure of 3DGNs, which could accumulate 1-NAP via π-π aggregation [19]. The enhanced signal could also be attributed to the excellent electric conductivity and large surface area of 3DGNs, which facilitate and accelerate electron transfer on the electrode interface [20].

Sensor performance was evaluated by DPV from the peak current of 0.35 V and with 1-NAP concentrations ranging from 10 nM to 10 μM. As shown in Figure 3b, the regression equation was y = 24.04x + 5.24, and the correlation coefficient *R*^2^ = 0.9985. The limit of detection (LOD) for 1-NAP was estimated to be 5.41 nM based on three times the standard deviation of the control sample (*n* = 3). The high sensitivity towards 1-NAP demonstrates the high electro-catalytic activity and potential of the 3DGNs-based sensor for subsequent ALP activity measurement.

### 3.3. Optimization of ALP Activity Detection

**Figure 4:**
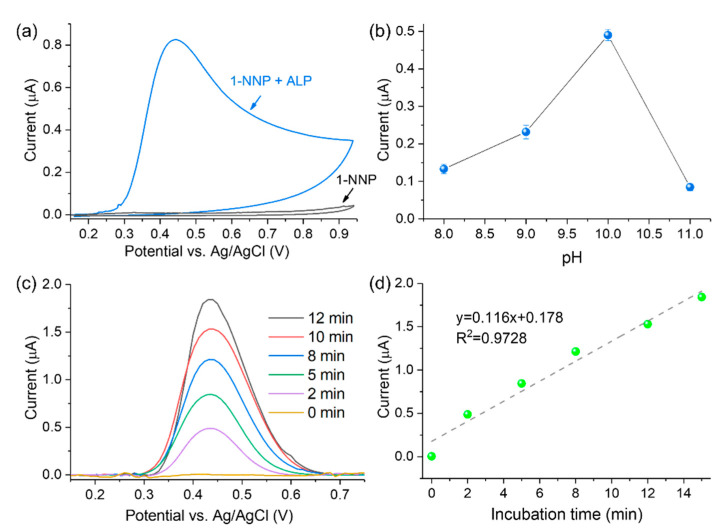
(**a**) CV test of ALP activity with 1-NPP as substrate; (**b**) pH optimization for the ALP activity detection (*n* = 3); (**c**) optimization of the incubation time for the ALP activity test (ALP activity is 100 U/L, and 2 M phosphoric acid used as stop solution); (**d**) linear relationship between the DPV current and incubation time.

In this work, the ALP activity was measured with the strategy illustrated in Figure 1. First, the substrate 1-NPP is enzymatically converted to 1-NAP by ALP. Then 1-NAP is oxidized to 1,4-naphthoquinone on the 3DGNs material, producing an amplified current response. As shown in Figure 4a, the sensor’s ability to detect ALP was investigated by CV. In the absence of ALP (black curve), no oxidation peaks were observed for 1-NPP, while an oxidation peak at 0.42 V appeared in the presence of ALP (blue curve). The results indicate that 1-NPP is non-electroactive and could not be hydrolyzed to 1-NAP without ALP. 

In order to obtain the optimal experimental conditions affecting the enzymatic reaction of ALP, the pH value and incubation time were optimized. It is known that ALP converts 1-NPP to 1-NAP under an alkaline environment [14]. Therefore, the pH optimum during ALP activity analysis was investigated from 8.0 to 11.0. As shown in Figure 4b, ALP offers the highest hydrolysis ability at pH = 10, which agrees with previous literature [32,33]. Hence, pH 10.0 was utilized for subsequent experiments. 

The effects of incubation time on sensor signal responses were investigated by the addition of a stop solution which can end the enzymatic reaction after a particular time [16]. Regarding the optimization of incubation time, 500 μM of 1-NPP substrate reacted with ALP (100 U/L), and 2 M phosphoric acid was added as the stop solution at different reaction periods. As shown in Figure 4c,d, the DPV peak current linearly increased with the incubation time. A current signal with a decent signal-to-noise ratio can be obtained in 2 min with the help of 3DGNs. Despite the stronger current and better sensor sensitivity, a longer incubation time prolongs the tests. Therefore, an incubation time of 2 min was chosen to establish a quick ALP activity detection protocol.

### 3.4. Detection of ALP Activity

**Figure 5:**
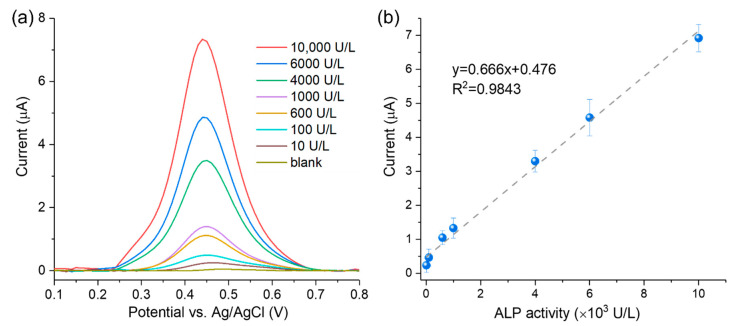
(**a**) DPV curves obtained from a series of ALP activities by 3DGNs/SPE; (**b**) calibration curve for ALP activity quantification (*n* = 3).

The sensitivity of the 3DGNs-based sensor was investigated by DPV. As shown in Figure 5a, the peak current of DPV increased with rising ALP activities on the optimized experimental conditions. Figure 5b exhibits the linear relationship between the DVP peak current and ALP activities from 10 to 10,000 U/L. The linear regression equation is y = 0.666x + 0.476 with a correlation coefficient *R*^2^ = 0.9843 (*n* = 3). The estimated LOD is 5.70 U/L, and the quantification limit was calculated as 20.67 U/L (ten times the standard deviation of the control sample). This sensitivity can satisfy the requirement for ALP activity detection in biological samples (46–190 U/L for adults [34]).

As shown in Table S1 (Supplementary Materials), our 3DGNs-based sensor exhibited good sensing performance, and the sensitivity was comparable to the reported techniques. In addition, our 3DGN-based sensor for ALP detection has the advantages of short assay time and wide detection range, which can be attributed to the signal amplification of 3DGNs for ALP hydrolyzed product, showing potential for real biological sample testing.

### 3.5. Detection of ALP in the Living Adherent Osteoblastic Cells Supernatant 

**Figure 6:**
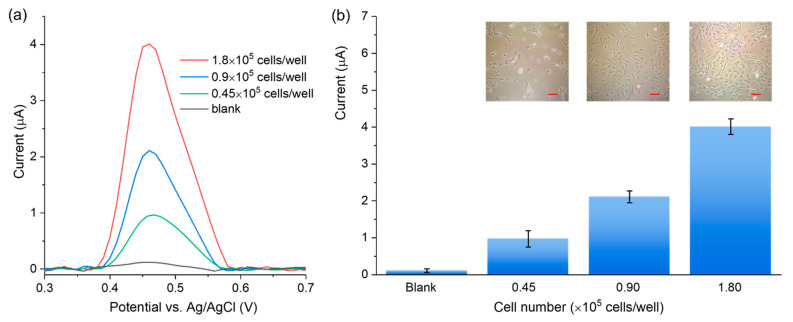
(**a**) DVP curves obtained from the supernatant of a series of IDG-SW3 cell densities; (**b**) the DPV signal increases with the rising cell densities, and the insets show optical images of the IDG-SW3 cells in the differentiation stage with different densities (scale bar = 25 μm).

The feasibility of the 3DGNs-based sensor was validated by measuring the activity of ALP secreted from the IDG-SW3 cells in the differentiation stage. IDG-SW3 cells were incubated for 1 day at various seeding densities (0.45 × 10^5^, 0.90 × 10^5^, and 1.80 × 10^5^ per well, respectively) for differentiation. Then the ALP activity was measured from the cell culture supernatant. As shown in Figure 6, the DPV peak current increased with the rising cell densities. The results demonstrate that the 3DGNs-based sensor can detect ALP released by adherent living cells in the culture supernatant. The incubation time of 2 min and a sample volume of 5 μL can guarantee the desired sensitivity for different cell densities. Moreover, our approach is non-destructive to cells, which makes it suitable for the long-time osteoblastic activity monitoring by analyzing the ALP activity in the supernatant. Since the cell density range is commonly used in the culture of osteogenic cells, the electrochemical method is also promising for measuring the ALP activity of other osteogenic cells.

## 4. Conclusions

In summary, 3DGNs nanostructure was successfully synthesized in-situ and used as a signal amplifier for electrochemical detection of ALP activity in both solution and cell supernatant. The unique porous structure and large surface area of 3DGNs enhance the synergetic effects from the π–π conjugate and electrochemical accumulation for ALP enzymatic product at the sensing interface, resulting in an amplified current signal. This sensor exhibits a LOD of 5.70 U/L in a wide range of ALP activities from 10 to 10,000 U/L. To measure ALP activity existing in the complex biological samples at trace levels, unlike some clinically used methods which require expensive and bulky instruments, complex pre-treatment procedures, and long assay times, the proposed micro-sensor exhibits high sensitivity, low sample consumption (5 μL) and a short incubation time (2 min). This 3DGNs-based electrochemical sensor is cost-effective, portable, disposable, and easy to operate, making it suitable for point-of-care testing (POCT) applications. Moreover, it detects cell-secreted ALP in a non-destructive way, having the potential for long-time osteoblastic activity monitoring in biomedical and clinical research.

## Data Availability

Not applicable.

## Supplementary Materials

The following supporting information can be downloaded at: https://www.mdpi.com/article/10.3390/bios12060406/s1, Table S1: Limit of detection of the proposed ALP sensor compared with other technologies [17,35,36,37,38,39,40,41,42].
