# Supplementary material for: Alkaline Phosphatase Electrochemical Micro-Sensor Based on 3D Graphene Networks for the Monitoring of Osteoblast Activity
Source: Biosensors (Basel). 2022 Jun 13;12(6):406. doi: 10.3390/bios12060406 (PMC9221009; doi:10.3390/bios12060406)
Supplement: Supplementary file 1 [file biosensors-12-00406-s001.zip › biosensors-1754729-supplementary.pdf]

Biosensors
Electronic Supplementary Information

Alkaline Phosphatase Electrochemical Micro-Sensor Based on 3D Graphene Networks for the Monitoring of Osteoblast Activity

S1. Surfece area determination

The specific surface area is measured by using the method based on UV-Vis absorption with methylene blue (MB) dye as probes [1]. 3DGNs were in-situ synthesized on PI substrate by a laser direct-writing technology in a square pattern (1 mm × 1mm), then 3DGNs was carefully collected by a scalpel. Next, 0.5 mg/mL of synthesized 3DGNs were mixed with 1 mg/mL MB in DI water. After gently stirred for 24 h, the 3DGNs were removed by centrifugation (12000 rpm, 20 min). The MB concentrations in water before and after adsorption were determined by UV–vis measurements at 445 nm (Jasco V-670 spectrophotometer). Then the adsorbed MB amount can be calculated from the UV–vis absorbance difference before and after the stirring (mg/g material). According to the previous reports [2,3], the surface covered per mg of adsorbed MB is 2.54 m^2. Using this method, the surface area of 3DGNs is measured to be 448.73 m^2/g.

Table S1. Limit of detection of the proposed ALP sensor compared with other technologies

Material	Method	Assay Time	LOD	Dynamic Range	Ref.
Gold nanoparticle	Colorimetric	1 min	0.16 U/20 μL	0.16-10 U/20 μL	[4]
Silver nanoparticle	Colorimetric	20 min	1 U/mL	1-100 U/mL	[5]
Alkynylplatinum (II) terpyridine complex	Fluorescence	Real time monitoring	8 U/L	10-1000 U/L	[6]
3,4-diaminobenzene-thiol modified gold nanoparticle	SERS	80 min	2 U/L	0.5-10 U/L	[7]
Nanoceria Particles	Electrochemistry	2 min	0.02 U/mL	5-640 U/mL	[8]
Au-nano-Dendroids and GO nanocomposite	Electrochemistry	30 min	9.10 U/L	100–1000 U/L	[9]
3DGNs	Electrochemistry	2 min	5.70 U/L	10-10000U/L	This work

References

1. Boulanger, N.; Kuzenkova, A.S.; Iakunkov, A.; Romanchuk, A.Y.; Trigub, A.L.; Egorov, A.V.; Bauters, S.; Amidani, L.; Retegan, M.; Kvashnina, K.O.; et al. Enhanced Sorption of Radionuclides by Defect-Rich Graphene Oxide. *ACS Appl. Mater. Interfaces* **2020**, *12*, 45122-45135, doi:10.1021/acsami.0c11122.
2. McAllister, M.J.; Li, J.-L.; Adamson, D.H.; Schniepp, H.C.; Abdala, A.A.; Liu, J.; Herrera-Alonso, M.; Milius, D.L.; Car, R.; Prud'homme, R.K.; et al. Single Sheet Functionalized Graphene by Oxidation and Thermal Expansion of Graphite. *Chem. Mater.* **2007**, *19*, 4396-4404, doi:10.1021/cm0630800.
3. Montes-Navajas, P.; Asenjo, N.G.; Santamaría, R.; Menéndez, R.; Corma, A.; García, H. Surface Area Measurement of Graphene Oxide in Aqueous Solutions. *Langmuir* **2013**, *29*, 13443-13448, doi:10.1021/la4029904.
4. Zhao, W.; Chiuman, W.; Lam, J.C.F.; Brook, M.A.; Li, Y. Simple and rapid colorimetric enzyme sensing assays using non-crosslinking gold nanoparticle aggregation. *ChemComm.* **2007**, 36, 3729-3731,doi:10.1039/b705335e.
5. Wei, H.; Chen, C.; Han, B.; Wang, E. Enzyme Colorimetric Assay Using Unmodified Silver Nanoparticles. *Anal. Chem.* **2008**, *80*, 7051-7055, doi:10.1021/ac801144t.
6. Yeung, M.C.-L.; Yam, V.W.-W. Phosphate derivative-induced supramolecular assembly and NIR-emissive behaviour of alkynylplatinum(ii) terpyridine complexes for real-time monitoring of enzymatic activities. *Chem. Sci.* **2013**, *4*, doi:10.1039/c3sc50383f.
7. Wang, W.; Zhang, Y.; Zhang, W.; Liu, Y.; Ma, P.; Wang, X.; Sun, Y.; Song, D. A novel sensing platform for the determination of alkaline phosphatase based on SERS-fluorescent dual-mode signals. *Anal. Chim. Acta* **2021**, *1183*, doi:10.1016/j.aca.2021.338989.
8. Hayat, A.; Andreescu, S. Nanoceria Particles As Catalytic Amplifiers for Alkaline Phosphatase Assays. *Anal. Chem.* **2013**, *85*, 10028-10032, doi:10.1021/ac4020963.
9. Mahato, K.; Purohit, B.; Kumar, A.; Chandra, P. Clinically comparable impedimetric immunosensor for serum alkaline phosphatase detection based on electrochemically engineered Au-nano-Dendroids and graphene oxide nanocomposite. *Biosens. Bioelectron.* **2020**, *148*, 111815, doi:10.1016/j.bios.2019.111815.
